# Involvement of Dual Strands of *miR-143* (*miR-143-5p* and *miR-143-3p*) and Their Target Oncogenes in the Molecular Pathogenesis of Lung Adenocarcinoma

**DOI:** 10.3390/ijms20184482

**Published:** 2019-09-11

**Authors:** Hiroki Sanada, Naohiko Seki, Keiko Mizuno, Shunsuke Misono, Akifumi Uchida, Yasutaka Yamada, Shogo Moriya, Naoko Kikkawa, Kentaro Machida, Tomohiro Kumamoto, Takayuki Suetsugu, Hiromasa Inoue

**Affiliations:** 1 Department of Pulmonary Medicine, Graduate School of Medical and Dental Sciences, Kagoshima University, Kagoshima 890-8520, Japan; 2 Department of Functional Genomics, Graduate School of Medicine, Chiba University, Chuo-ku, Chiba 260-8670, Japan; 3 Department of Biochemistry and Genetics, Graduate School of Medicine, Chiba University, Chuo-ku, Chiba 260-8670, Japan

**Keywords:** lung adenocarcinoma, microRNA, *miR-143-5p*, *miR-143-3p*, tumor-suppressor, *MCM4*

## Abstract

Our analyses of tumor-suppressive microRNAs (miRNAs) and their target oncogenes have identified novel molecular networks in lung adenocarcinoma (LUAD). Moreover, our recent studies revealed that some passenger strands of miRNAs contribute to cancer cell malignant transformation. Downregulation of both strands of the *miR-143* duplex was observed in LUAD clinical specimens. Ectopic expression of these miRNAs suppressed malignant phenotypes in cancer cells, suggesting that these miRNAs have tumor-suppressive activities in LUAD cells. Here, we evaluated *miR-143-5p* molecular networks in LUAD using genome-wide gene expression and miRNA database analyses. Twenty-two genes were identified as potential *miR-143-5p*-controlled genes in LUAD cells. Interestingly, the expression of 11 genes (*MCM4*, *RAD51*, *FAM111B*, *CLGN*, *KRT80*, *GPC1*, *MTL5*, *NETO2*, *FANCA*, *MTFR1*, and *TTLL12*) was a prognostic factor for the patients with LUAD. Furthermore, knockdown assays using siRNAs showed that downregulation of *MCM4* suppressed cell growth, migration, and invasion in LUAD cells. Aberrant expression of *MCM4* was confirmed in the clinical specimens of LUAD. Thus, we showed that *miR-143-5p* and its target genes were involved in the molecular pathogenesis of LUAD. Identification of tumor-suppressive miRNAs and their target oncogenes may be an effective strategy for elucidation of the molecular oncogenic networks of this disease.

## 1. Introduction

Lung cancer (non-small cell lung cancer (NSCLC) and small cell lung cancer (SCLC)) is the leading cause of cancer-related deaths among men and women in developed countries, including Japan. In 2018, approximately 2,300,000 people were diagnosed with lung cancer, and 1,800,000 patients died from this disease [1]. NSCLC (accounting for approximately 80% of lung cancer cases) is divided mainly into three subtypes: lung adenocarcinoma (LUAD), squamous cell carcinoma (LUSQ), and large cell carcinoma [2]. Approximately 40% of patients with lung cancer have LUAD, and this type of lung cancer is often observed in patients (especially young women) who have never smoked, in contrast to other types of lung cancers [3].

LUAD has been extensively studied using molecular biology analyses, and various treatment options have been developed as a result. Based on the mutation statuses of cancer cells, several molecular targeted drugs have been available (e.g., epidermal growth factor receptor tyrosine kinase inhibitors and anaplastic lymphoma kinase inhibitors), and these treatments have dramatically improved patient outcomes [4,5]. However, these treatment effects are limited because acquired resistance and cancer recurrence occur in almost all patients [6].

Human genome research has revealed that the human genome encodes a large number of protein-noncoding RNA molecules, which are transcribed and have various important functions [7]. microRNAs (miRNAs) are small, noncoding RNAs that act as fine-tuners of RNA expression in cells [8]. Notably, bioinformatics predictions have shown that a single miRNA can control a vast number of RNA transcripts in cells, and more than half of RNAs in the human genome are controlled by miRNAs [9]. The search for molecular networks controlled by miRNAs has provided us with novel knowledge of RNA networks in normal and diseased cells.

Aberrantly expressed miRNAs disrupt RNA networks and enhance cancer cell aggressive phenotypes (e.g., development, metastasis, and drug resistance) [10]. Identification of tumor-suppressive or oncogenic miRNAs and their target cancer-related genes has contributed to understanding of the molecular pathogenesis of human cancers [11]. Based on miRNA expression signatures, we have sequentially identified antitumor miRNAs and their target oncogenes in several cancers [12,13,14,15].

In the conventional miRNA biogenesis process, the guide strand of miRNA derived from the miRNA duplex functions to control the target molecules [16], whereas the passenger strand of miRNA is not incorporated into the RNA-induced silencing complex (RISC), and it is disassembled and has no function [17]. However, in lung cancer, including SCLC, LUAD, and LUSQ, our recent studies showed that both strands of the miRNA duplex (e.g., *miR-144-5p/-3p* in LUSQ, *miR-145-5p*/*-3p* in LUSQ and LUAD, and *miR-150-5p*/*-3p* in LUAD) are significantly downregulated in lung cancer tissues, and these miRNAs act as tumor-suppressive miRNAs by targeting several oncogenes [18,19,20,21]. Moreover, the expression levels of miRNAs and their target genes are closely associated with lung cancer pathogenesis [22].

In this study, we focused on both strands of the *miR-143* duplex (*miR-143-5p*: the passenger strand, and *miR-143-3p*: the guide strand) in LUAD cells. Previous studies have shown that *miR-143-3p* functions as a tumor-suppressive miRNA in many types of cancers, and the oncogenes regulated by this miRNA duplex were identified [23,24]. On the other hand, functional analysis of *miR-143-5p* in cancer cells has not previously been reported. Here, we showed that ectopic expression of *miR-143-5p* attenuated malignant phenotypes in LUAD cells. Furthermore, 11 genes (*MCM4*, *RAD51*, *FAM111B*, *CLGN*, *KRT80*, *GPC1*, *MTL5*, *NETO2*, *FANCA*, *MTFR1*, and *TTLL12*) were found to be closely associated with the molecular pathogenesis of LUAD. Identification of tumor-suppressive miRNAs and their oncogenes may be an effective strategy for elucidation of the molecular pathogenesis of this disease.

## 2. Results

### 2.1. Downregulation of the miR-143 Duplex (miR-143-5p and miR-143-3p) in LUAD Clinical Specimens and Cell Lines

**Figure 1:**
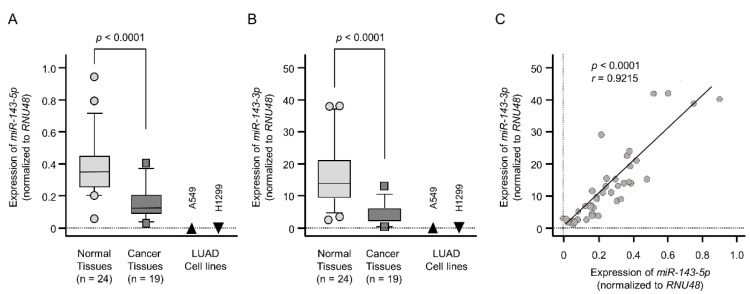
Expression of *miR-143-5p* and *miR-143-3p* in LUAD clinical specimens and cell lines. (**A**) Downregulation of *miR-143-5p* was detected in LUAD specimens and cell lines (A549 and H1299 cells). Data were normalized according to the expression of *RNU48* (internal control). (**B**) Downregulation of *miR-143-3p* was detected in clinical specimens and cell lines. (**C**) *miR-143-5p* and *miR-143-3p* expression levels were positively correlated in clinical specimens, as demonstrated by Spearman’s rank tests.

Expression levels of *miR-143-5p* and *miR-143-3p* were markedly reduced in cancer tissues compared with those in normal tissues (*p* < 0.0001 and *p* < 0.0001, respectively; Figure 1A,B). The clinical features of LUAD specimens are listed in Table 1 and Table S1. In two LUAD cell lines (A549 and H1299), the expression levels of these miRNAs were very low compared with those in normal tissues (Figure 1A,B). A positive correlation was detected between *miR-143-5p* and *miR-143-3p* expression levels by Spearman’s rank analysis (*r* = 0.9215, *p* < 0.0001; Figure 1C).

Cohort analysis using data from The Cancer Genome Atlas (TCGA) database showed that there was no significant relationship between expression of *miR-143-5p* or *miR-143-3p* and prognosis of patients with LUAD (Figure S1).

### 2.2. Ectopic Expression of miR-143-5p and miR-143-3p Blocked LUAD Aggressiveness

**Figure 2:**
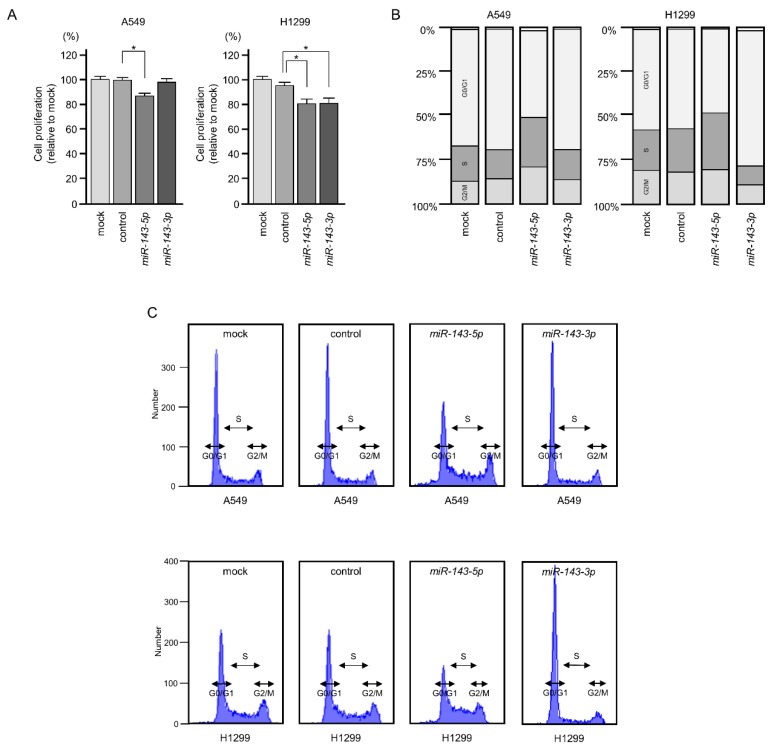
Cell proliferation and cell cycle assays in LUAD cell lines with ectopic expression of *miR-143-5p* and *miR-143-3p*. (**A**) Cell proliferation was assessed using XTT assays. Data were measured 72 h after transfection with miRNAs. Cell proliferation was suppressed by *miR-143-5p* and *miR-143-3p* in H1299 cells (* *p* < 0.001), but it was not affected by *miR-143-3p* transfection into A549 cells. (**B**,**C**) Cell cycle assays by flow cytometry showed that G_2_/M phase arrest was induced following *miR-143-5p* transfection into A549 and H1299 cells. In contrast to *miR-143-5p* transfection, G_0_/G_1_ phase arrest was detected by *miR-143-3p* transfection.

To investigate the functions of *miR-143-5p* and *miR-143-3p* in LUAD cells, we designed ectopic expression assays to assess cell proliferation, migration, and invasion after transfection of these miRNAs into A549 and H1299 cells. Gain-of-function assays showed that overexpression of *miR-143-5p* attenuated cell proliferation in A549 and H1299 cells (Figure 2A). However, *miR-143-3p* expression did not affect cell proliferation in A549 cells (Figure 2A). We counted LUAD cells for the antiproliferative effects of ectopic expression of *miR-143-5p* and *miR-143-3p*, and obtained similar results with the XTT assay (Figure S2). Cell cycle distributions were analyzed by flow cytometry following transfection of LUAD cells with *miR-143-5p* and *miR-143-3p* (72 h after transfection). Cell cycle phase distributions (G_0_/G_1_, S, and G_2_/M) are shown in the bar chart. G_2_/M phase arrest was detected by *miR-143-5p* transfection into LUAD cells. In contrast to *miR-143-5p* transfection, G_0_/G_1_ phase arrest was detected by *miR-143-3p* transfection (Figure 2B,C).

**Figure 3:**
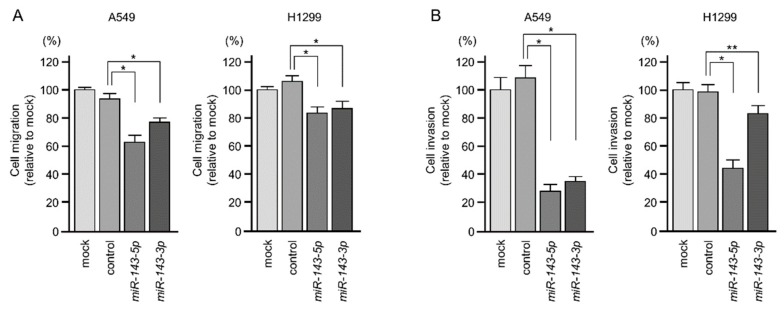
Cell migration and invasion assays in LUAD cells with ectopic expression of *miR-143-5p* and *miR-143-3p*. Cells were transfected with *miR-143-5p* and *miR-143-3p*. (**A**) Cell migration was measured (48 h after wounding the cells) by wound healing assays (* *p* < 0.001). (**B**) Cell invasion was determined (48 h after seeding cells into the chamber) by Matrigel invasion assays (* *p* < 0.001, ** *p* < 0.05).

Moreover, as shown in Figure 3A,B, the migration and invasion of A549 and H1299 cells were significantly suppressed by *miR-143-5p* or *miR-143-3p* transfection. The photomicrographs are presented in Figure S3.

Due to the cell migration and invasive abilities being remarkably suppressed by *miR-143-5p* and *miR-143-3p* expression in LUAD cells, we investigated the regulation of epithelial–mesenchymal transition (EMT)-related genes (e.g., *E*-cadherin, Vimentin, *N*-cadherin, SNAIL, and SLUG) by these miRNAs. Interestingly, the expression of Vimentin, SNAIL, and SLUG was reduced by *miR-143-5p* in LUAD cells (Figure S4). The elucidation of the molecular mechanism of suppression of these EMT-related genes by *miR-143-5p* is an issue for the future.

### 2.3. Incorporation of miR-143-5p into the RISC in LUAD Cells

To verify that *miR-143-5p* had actual functions in LUAD cells, we performed immunoprecipitation assays using anti-Ago2 antibodies. Ago2 is an essential component of the RISC. After transfection of *miR-143-5p* into A549 cells, the amount of *miR-143-5p* was dramatically increased compared with that in untransfected cells (Figure S5). These data suggested that transfected *miR-143-5p* was incorporated into the RISC in LUAD cells.

### 2.4. Screening of miR-143-5p-Controlled Oncogenes in LUAD Cells

To identify genes controlled by *miR-143-5p* in LUAD cells, we used three independent datasets: gene expression data from *miR-143-5p*-transfected A549 cells, gene expression data from lung cancer clinical specimens, and data from the TargetScanHuman database, showing annotated putative targets with *miR-143-5p* binding sites. Our strategy searching for *miR-143-5p* targets is shown in Figure S6. In this study, 22 genes were identified as putative *miR-143-5p*-controlled oncogenes in LUAD cells (Table 2). We also identified *miR-143-3p*-controlled oncogenic targets using a similar strategy (Figure S6, Table S2).

### 2.5. Clinical Significance of miR-143-5p Targets in LUAD Pathogenesis

**Figure 4:**
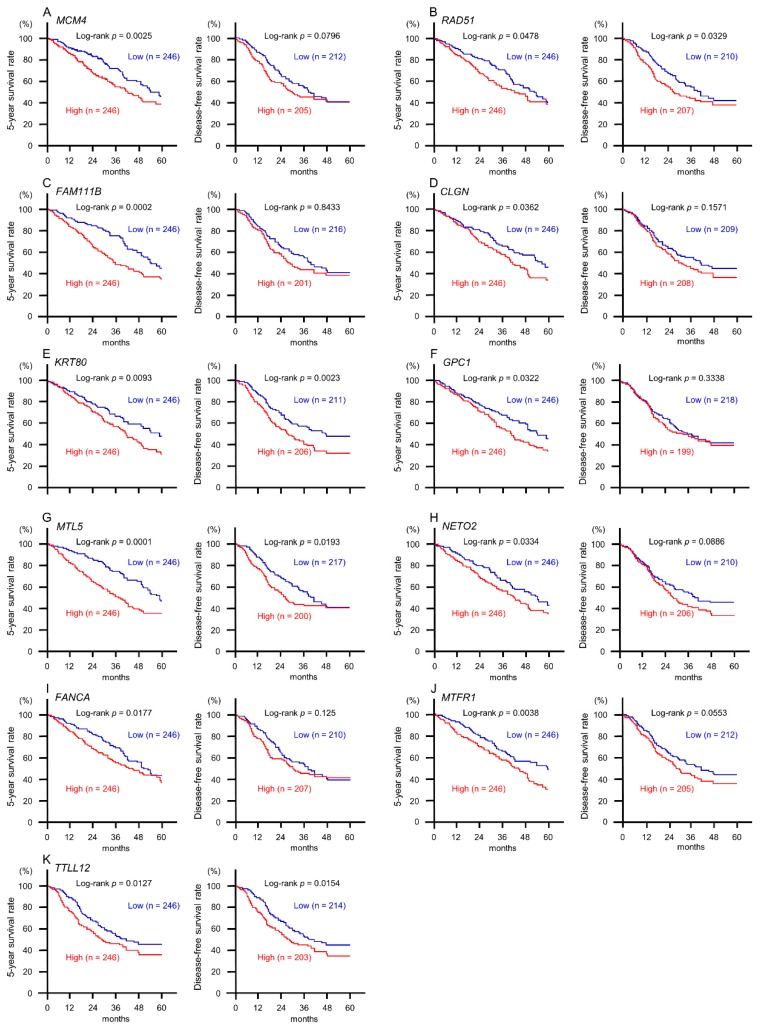
Clinical significance of *miR-143-5p* target genes in TCGA database. Among putative targets of *miR-143-5p* in LUAD cells, high expression of 11 genes (*MCM4*, *RAD51, FAM111B*, *CLGN*, *KRT80*, *GPC1*, *MTL5*, *NETO2*, *FACNCA*, *MTFR1*, and *TTLL12*) was significantly associated with poor prognosis in patients with LUAD. Kaplan–Meier curves of 5-year overall survival and 5-year disease-free survival are shown.

**Figure 5:**
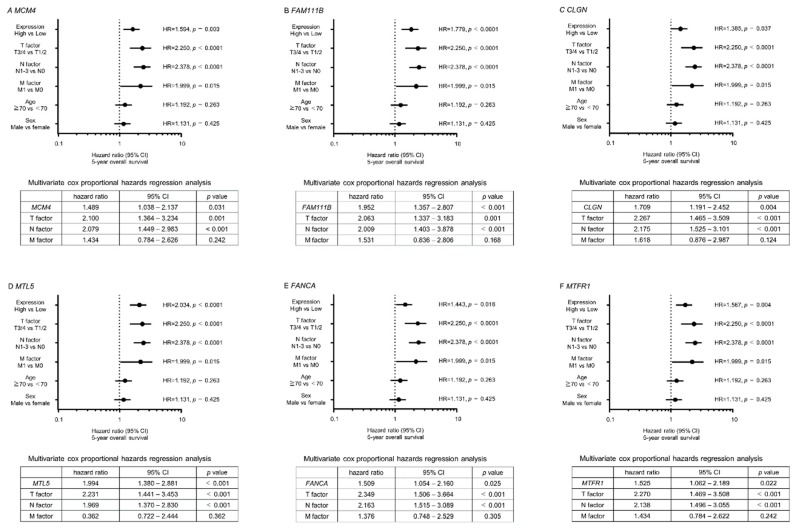
Multivariate analysis of six genes (*MCM4*, *FAM111B*, *CLGN*, *MTL5*, *FANCA*, and *MTFR1*). These genes were independent prognostic factors for overall survival.

Next, we investigated the clinical significance of the 22 target genes in the pathogenesis of LUAD by using TCGA database. Among the 22 targets, high expression of 11 genes (*MCM4*, *RAD51*, *FAM111B, CLGN*, *KRT80*, *GPC1*, *MTL5*, *NETO2*, *FANCA*, *MTFR1*, and *TTLL12*) was closely associated with poor prognosis (overall survival rate: *p* < 0.05) in patients with LUAD (Table 2 and Figure 4). Interestingly, multivariate analysis revealed that expression levels of six genes (*MCM4*, *FAM111B*, *CLGN*, *MTL5*, *FANCA*, and *MTFR1*) were independent predictive factors for overall survival rate in these patients (Figure 5).

**Figure 6:**
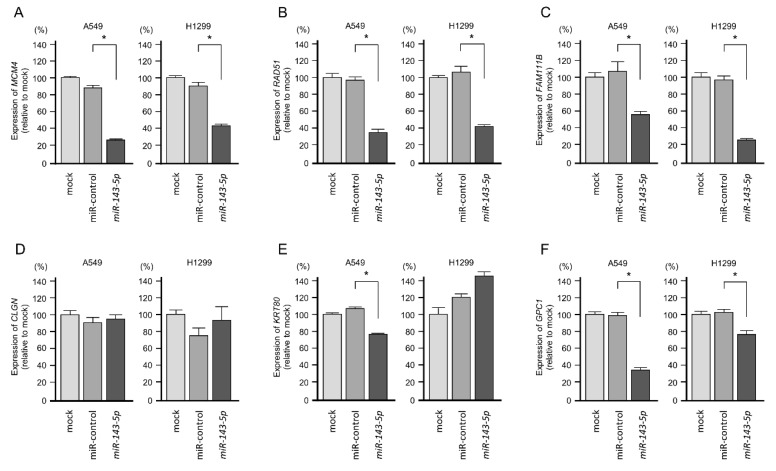

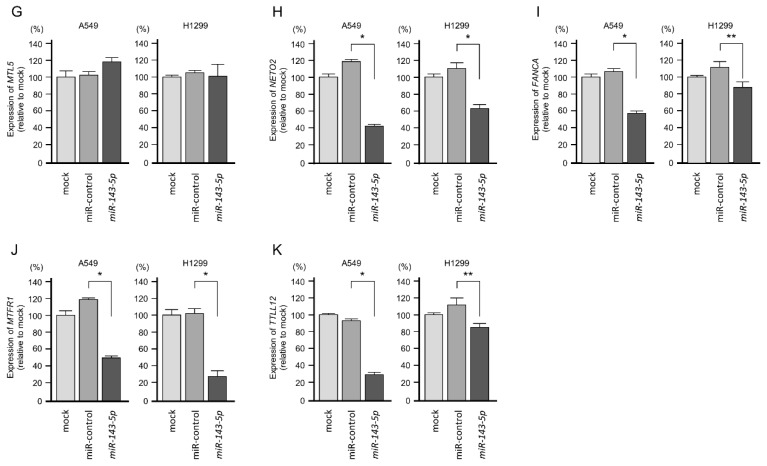
Expression of *miR-143-5p* target genes was evaluated using quantitative real-time reverse transcription polymerase chain reaction (qRT-PCR). (**A**) *MCM4*, (**B**) *RAD51*, (**C**) *FAM111B*, (**D**) *CLGN*, (**E**) *KRT80*, (**F**) *GPC1*, (**G**) *MTL5*, (**H**) *NETO2*, (**I**) *FANCA*, (**J**) *MTFR1*, (**K**) *TTLL12*. Expression of *GAPDH* was used as an internal control (* *p* < 0.001, ** *p* < 0.05).

We then examined the regulation of these 11 genes by *miR-143-5p* in LUAD cells. We confirmed that eight genes (*MCM4*, *RAD51*, *FAM111B*, *GPC1*, *NETO2*, *FANCA*, *MTFR1*, and *TTLL12*) were downregulated in *miR-143-5p* transfected in A549 and H1299 cells (Figure 6).

### 2.6. Direct Regulation of MCM4 Expression by miR-143-5p in LUAD Cells

From our present data (Table 2, Figure 4, Figure 5 and Figure 6), it has become clear that the four genes (*MCM4*, *FAM111B*, *FANCA,* and *MTFR1*) are under the control of antitumor *miR-143-5p* and are involved in the malignant transformation of LUAD.

**Figure 7:**
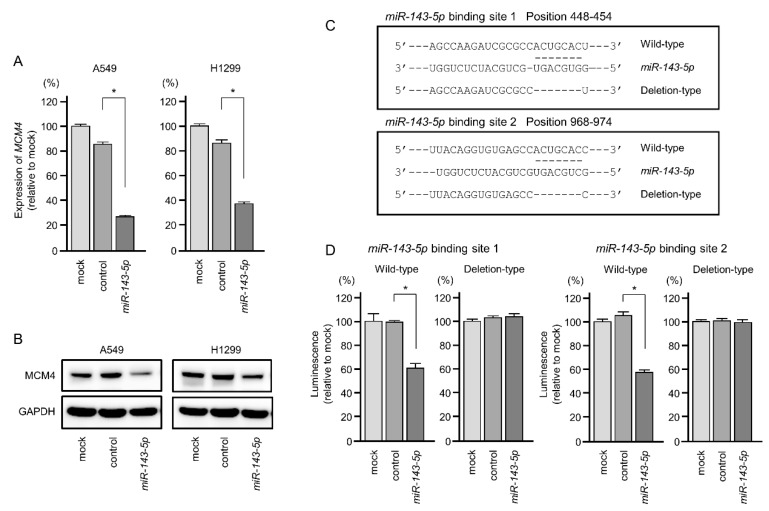
Expression of *MCM4*/MCM4 was directly regulated by *miR-143-5p* in LUAD cells. (**A**) Expression of *MCM4* mRNA was significantly reduced by *miR-143-5p* transfection into A549 and H1299 cells (72 h after transfection). Expression of *GAPDH* was used as an internal control. (**B**) Expression of MCM4 protein was reduced by *miR-143-5p* transfection into LUAD cells (72 h after transfection). Expression of GAPDH was used as an internal control. (**C**) TargetScanHuman database analyses predicted two putative *miR-143-5p* binding sites in the 3′-UTR of *MCM4*. (**D**) Dual luciferase reporter assays showed that luminescence activities were reduced by cotransfection with wild-type (*miR-143-5p* binding site) vectors and *miR-143-5p* in LUAD cells. Normalized data were calculated as *Renilla*/firefly luciferase activity ratios (* *p* < 0.001).

We focused on further evaluation of *MCM4* in LUAD cells. Expression levels of *MCM4* mRNA and MCM4 protein were significantly reduced by *miR-143-5p* transfection in A549 and H1299 cells (Figure 7A,B).

Next, we performed dual-luciferase reporter assays to determine whether *MCM4* was directly regulated by *miR-143-5p*. We used vectors encoding the partial wild-type sequences of the 3′- untranslated region (UTR) of *MCM4* including the predicted *miR-143-5p* target sites (binding site 1: positions 448–454, binding site 2: positions 968–974 in the *MCM4* 3′-UTR) or deletion vectors lacking these sites (Figure 7C). We found that the luciferase activity was significantly decreased by cotransfection with *miR-143-5p* and the vector carrying the wild-type 3′-UTR of *MCM4*, whereas transfection with the deletion vector blocked the decrease in luminescence in A549 cells (Figure 7D). These data demonstrated that *miR-143-5p* directly bound to two sites in the 3′-UTR of *MCM4*.

### 2.7. Effects of MCM4 Knockdown on LUAD Cells Malignant Phenotypes

**Figure 8:**
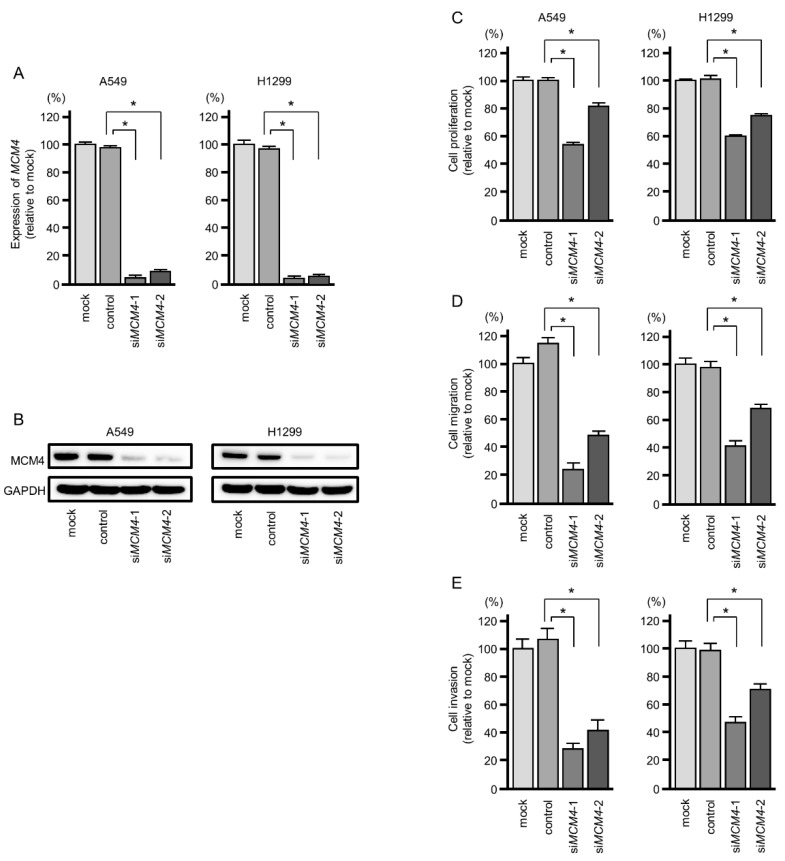
Effects of *MCM4* silencing on cell proliferation, migration, and invasion in LUAD cells. (**A**,**B**) The effects of si*MCM4* in LUAD cells. *MCM4* mRNA and protein expression levels were significantly reduced by si*MCM4* transfection into A549 and H1299 cells (72 h after transfection). Expression of *GAPDH*/GAPDH was used as an internal control. (**C**) Cell proliferation activities were blocked by si*MCM4* transfection into LUAD cells. (**D**,**E**) Cell migration and invasive abilities were blocked by si*MCM4* transfection into LUAD cells (* *p* < 0.001).

To investigate the oncogenic functions of *MCM4* in LUAD cells, knockdown assays were conducted using small interfering RNAs (siRNAs). Both mRNA and protein expression levels were successfully suppressed by si*MCM4* transfection in A549 and H1299 cells (Figure 8A,B).

In functional assays, cell proliferation was significantly suppressed by si*MCM4* transfection into A549 and H1299 cells (Figure 8C). Cell migration and invasive abilities were significantly blocked by knockdown of *MCM4* (si*MCM4*-1 and si*MCM4*-2) in LUAD cells (Figure 8D,E). The photomicrographs were presented in Figure S7. Cell cycle assays showed that significant G_0_/G_1_ phase arrest was observed in LUAD cells (Figure S8). In functional assays, cell proliferation was significantly suppressed by si*MCM4* transfection into A549 and H1299 cells (Figure 8C). Moreover, cell migration and cell invasion were significantly suppressed by si*MCM4* transfection into A549 and H1299 cells (Figure 8D,E). Notably, cell cycle assays by *MCM4* knockdown showed that significant G_0_/G_1_ phase arrest was observed in LUAD cells (Figure S8). Ectopic expression of *miR-143-5p* induced the S phase arrest or delayed the S phase progression (Figure 2). *MCM4* is one of the pivotal genes directly controlled by *miR-143-5p* in LUAD cells. This suggests that *miR-143-5p* also regulates other cell-cycle-related genes involved in S phase progression.

### 2.8. Aberrant Expression of MCM4 in LUAD Clinical Specimens by Immunohistochemistry

**Figure 9:**
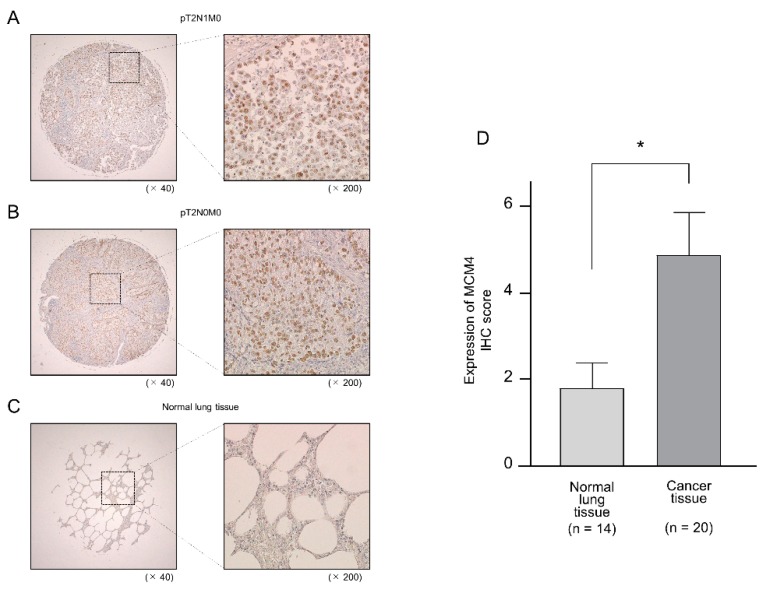
Aberrant expression of MCM4 in LUAD clinical specimens. Patient characteristics showed in Table S3. (**A**–**C**) Expression of MCM4 was investigated by immunohistochemistry staining of LUAD specimens and normal lung tissues. Overexpression of MCM4 was detected in nuclei of cancer lesions, whereas negative or low expression of MCM4 was observed in normal lung tissue (**A**; patient no.16, **B**; patient no.19, **C**; patient no.31). (**D**) Comparison of the scoring of MCM4 expression in clinical lung specimens. Expression scores of MCM4 in LUAD tissues were significantly higher than those in normal lung tissue (* *p* = 0.0414).

MCM4 expression at the protein levels were evaluated by using tissue microarray of 20 LUAD cases and 14 noncancerous cases (Table S3). Compared with normal lung tissues, MCM4 protein was strongly expressed in LUAD tissues (Figure 9A–D).

We also investigated the expression of MCM4 using *miR-143-5p*-measured clinical specimens. Overexpression of MCM4 was detected in LUAD clinical specimens (Table S1 and Figure S9).

## 3. Discussion

Several miRNAs exist in close proximity on the human genome (defined as miRNA clusters). The expression levels of miRNAs within miRNA clusters are controlled by the same molecular mechanisms [25]. In the human chromosome 5q32 region, pre-*miR-143* and pre-*miR-145* form the miRNA cluster, and their promoter regions contain p53 binding sites, resulting in p53-dependent expression [26]. Many studies have shown that *miR-143*/*miR-145* cluster miRNAs are frequently downregulated in human cancers where *miR-143-3p* and *miR-145-5p* (the guide strand) act as pivotal tumor suppressors [27,28]. Extensive searches have been carried out to identify the oncogenic targets and molecular networks controlled by *miR-143-3p* and *miR-145-5p* in several cancers [27,28,29]. In contrast, few studies have evaluated functional roles and targets of *miR-143-5p* and *miR-145-3p* (the passenger strand) in cancer cells.

Recently, we found that the expression levels of both strands of pre-*miR-145* (*miR-145-5p* and *miR-145-3p*) were reduced in LUAD clinical specimens and that cancer cell malignant phenotypes were attenuated by ectopic expression of these miRNAs [20]. Furthermore, target searches for *miR-145-5p*/*miR-145-3p* showed that aberrant expression of *LMNB2* acted as an oncogene in LUAD cells [20]. Our previous studies revealed that tumor-suppressive *miR-145-3p* was closely involved in human cancer pathogenesis by targeting oncogenes, for example, *MTDH* in LUSQ; *MELK*, *NCAPG*, *BUB1*, and *CDK1* in prostate cancer; *MYO1B* in head and neck cancer; and *MYO1B* and *DHRS2* in esophageal cancer [19,30,31,32]. These data suggest that some passenger strands of miRNAs are actually functional in human cancers.

In this study, we focused on *miR-143-5p* and demonstrated the tumor-suppressive functions of this miRNA, similar to those of *miR-143-3p*, in LUAD cells. Previous studies have shown that *miR-143-5p* is downregulated in gastric cancer and gallbladder cancer tissues and that forced *miR-143-5p* expression suppresses cancer cell malignancy through targeting *COX-2* and *HIF1-alpha*, respectively [33,34]. Interestingly, recent reports have indicated that some long noncoding RNAs (e.g., *lncRNA-TCONS_00026907*, *ZEB2-AS1*, and *LINC01207*) suppress *miR-143-5p* expression to enhance cancer cell aggressiveness [35,36,37]. These findings were consistent with our current results in LUAD cells.

Next, we identified cancer-promoting genes and oncogenic molecular networks regulated by tumor-suppressive *miR-143-5p* in LUAD cells. In this screening, we successfully identified 22 putative target oncogenes regulated by *miR-143-5p* in LUAD cells. Among these targets, high expression of 11 genes (*MCM4*, *RAD51*, *FAM111B*, *CLGN*, *KRT80*, G*PC1*, *MTL5*, *NETO2*, *FANCA*, *MTFR1*, and *TTLL12*) was significantly associated with poor prognosis in patients with LUAD. Moreover, multivariate analysis revealed the expression levels of six genes (*MCM4*, *FAM111B*, *CLGN*, *MTL5*, *FANCA*, and *MTFR1*) were independent predictive factors for overall survival in patients. Recently, overexpression of *MTL5* was reported in NSCLC, and its expression was shown to predict prognosis [38]. Another study showed that knockdown of *FAM111B* induced G_2_/M phase arrest and apoptosis in LUAD cells [39]. These data suggested that *miR-143-5p*-controlled genes were closely involved in LUAD oncogenesis and molecular pathogenesis.

In this study, we further analyzed the roles of *MCM4* in LUAD cells. Our data revealed that aberrant expression of *MCM4* enhanced LUAD cell proliferation and that *MCM4* expression was an independent prognostic biomarker in patients with LUAD. Our data were consistent with a previous report [20], strongly suggesting that aberrantly expressed *MCM4* was an oncogene in LUAD.

Minichromosome maintenance (MCM) proteins (MCM2–MCM7) form a hexamer complex that functions as a DNA helicase in DNA replication [40]. Moreover, the MCM2-7 hexamer helicase is activated by forming a complex with CDC45 and GINS proteins, termed the CMG (CDC45/MCMs/GINS) complex [40]. Notably, TCGA database analyses showed that high expression of *CDC45*, *GINS1*, and *GINS3* was associated with a poor prognosis compared to low expression of these genes in patients with LUAD. Moreover, aberrant expression of MCM proteins has been shown to contribute to cancer progression, and these proteins could be effective diagnostic markers in various cancers [41]. In lung cancer, high expression levels of *MCM2*, *MCM5*, *MCM6*, and *MCM7* could be useful prognostic markers [41,42,43].

This is the first report demonstrating that tumor-suppressive *miR-143-5p* directly regulates *MCM4* in LUAD cells. However, our understanding of the molecular mechanisms controlling the expression of MCM proteins is insufficient. Recent studies have reported that tumor-suppressive miRNAs negatively regulate the expression of MCM family genes (e.g., *MCM2* is targeted by *miR-31* and *MCM5* is targeted by *miR-885-5p* and *miR-362-3p*) [44,45,46]. Interestingly, reduced expression of MCM2-7 in DNA replication stress is associated with p53-dependent miRNAs, including the miR-34-family [47]. Future studies are needed to determine the involvement of noncoding RNAs, including miRNAs, in the regulation of MCM/CMG expression.

Our results showed that all members of the *miR-143*/*miR-145* cluster (*miR-143-5p*, *miR-143-3p*, *miR-145-5p*, and *miR-145-3p*) acted as tumor-suppressive miRNAs in LUAD. The involvement of passenger strands of miRNAs in LUAD oncogenesis is a novel concept in cancer research. The tumor-suppressive functions of miRNAs depend on controlling a vast number of oncogenic genes in the cancer cell. Our present study suggests that the *miR-143-5p* regulates cell-cycle-related genes and EMT-related genes in LUAD cells. The *miR-143-5p* may control many genes contributing to LUAD malignancy. Identification of oncogenes and oncogenic networks controlled by the *miR-143*/*miR-145* cluster could help to establish novel prognostic and therapeutic targets in LUAD.

## 4. Materials and Methods

### 4.1. Clinical Human LUAD Specimens and LUAD Cell Lines

Forty-three clinical specimens (19 LUAD and 24 normal lung tissues) were used in this study. The clinical characteristics of these specimens are summarized in Table 1 and Table S1. Classification of sample stages was carried out according to the Association for the Study of Lung Cancer TNM classification (7th edition).

All specimens were obtained from lung cancer surgeries performed at Kagoshima University Hospital (2010–2013). Written informed consent was obtained from all patients before the use of their specimens. This study was approved by the Bioethics Committee of Kagoshima University (approval number: 26-164, 10th February 2015).

Two LUAD cell lines, A549 and H1299 (American Type Culture Collection, Manassas, VA, UAS), were used in this study.

### 4.2. RNA Preparation and Quantitative Real-Time Reverse Transcription Polymerase Chain Reaction (qRT-PCR)

RNA extraction from clinical specimens and cell lines was performed as previously described [21,48]. Expression levels of miRNAs were evaluated using qRT-PCR as described previously [21,48]. TaqMan probes and primers used in this study are listed in Table S4.

### 4.3. Transfection of miRNAs, siRNAs, and Plasmid Vectors into LUAD Cells

The procedures for transfection of miRNAs, siRNAs, and plasmid vectors into LUAD cells were described previously [21,48]. The reagents used in this study are listed in Table S4.

### 4.4. Functional Assays for LUAD Cells (i.e., Cell Proliferation, Migration, Invasion, and Cell Cycle Assays)

The procedures for functional assays in cancer cells (proliferation, migration, invasion, and cell cycle assays) were described in our previous studies [21,48].

### 4.5. Measurement of Amount of miR-143-5p Incorporated into the RISC

Immunoprecipitation using anti-Ago2 antibodies was used to determine whether *miR-143-5p* was incorporated into the RISC. The procedure for immunoprecipitation was described in a previous study [31]. The reagents used in this study are listed in Table S4.

### 4.6. Identification of Oncogenes Regulated by miR-143-5p and miR-143-3p in LUAD Cells

The strategy for identification of miRNA targets in this study is summarized in Figure S6. Three expression profiles (i.e., *miR-143-5p*-transfected A549 cells (GEO accession number: GSE123318), *miR-143-3p*-transfected A549 cells (accession number: GSE123318), and LUAD clinical specimens (accession number: GSE19188)), were used in this screening. TargetScanHuman database (http://www.targetscan.org/ver_72/) was used to predict miRNA binding sites.

### 4.7. Plasmid Construction and Dual-Luciferase Reporter Assay

Plasmid vectors, including vectors containing the wild-type sequence of *miR-143-5p* binding sites in the 3′-UTR of *MCM4* or the deletion sequences of *miR-143-5p* binding sites in the 3′-UTR of *MCM4*, were prepared. The inserted sequences are shown in Figure S10. The procedures for transfection and dual luciferase reporter assays were described in our previous studies [21,48]. The reagents used in this study are listed in Table S4.

### 4.8. Clinical Data Analyses of miRNAs and Target Genes in LUAD Specimens

TCGA (https://tcga-data.nci.nih.gov/tcga/) was applied to investigate the clinical significance of miRNAs and their target genes. Gene expression and clinical data were obtained from cBioPortal (http://www.cbioportal.org/) and OncoLnc (http://www.oncolnc.org/) (data downloaded on October 11, 2018).

### 4.9. Western Blotting and Immunohistochemistry

The procedures for Western blotting and immunohistochemistry were described in our previous studies [21,48]. MCM4 expression at the protein levels were evaluated by immunohistochemical staining using tissue microarray (BC04002a; US Biomax, Inc. Derwood, MD, USA) of 20 LUAD cases and 14 noncancerous cases (Table S3). The expression of MCM4 using *miR-143-5p*-measured clinical specimens was investigated (Table S1 and Figure S9). The antibodies used in this study are listed in Table S4.

### 4.10. Statistical Analysis

Mann–Whitney U tests were applied for comparisons between two groups. For multiple groups, one-way analysis of variance and Tukey tests for post hoc analysis were applied. These analyses were performed with GraphPad Prism 7 (GraphPad Software, La Jolla, CA, USA) and JMP Pro 14 (SAS Institute Inc., Cary, NC, USA).

## 5. Conclusions

This is the first report revealing that *miR-143-5p* (the passenger strand of the *miR-143* duplex) had tumor-suppressive functions in LUAD cells. Several genes controlled by *miR-143-5p* were closely involved in LUAD oncogenesis and pathogenesis. Notably, the expression of *MCM4* was directly regulated by *miR-143-5p*, and its aberrant expression enhanced LUAD malignant transformation. Our studies demonstrated that all members of the *miR-143*/*miR-145* cluster acted as tumor-suppressive miRNAs and that their targets may be novel prognostic and therapeutic targets in LUAD.

## Figures and Tables

**Table 1:** Characteristics of patients with LUAD and noncancerous controls.

A. Characteristics of Patients with LUAD		
**Patients with Lung Cancer**	**n**	(%)
Total number	19	
Median age (range)	73 (57–86)	
Sex		
Male	10	52.6
Female	9	47.4
Pathological stage		
IA	1	5.3
IB	4	21.0
IIA	9	47.4
IIB	1	5.3
IIIA	4	21.0
B. Characteristics of controls		
Controls	n	(%)
Total number	24	
Median age (range)	72.5 (50–88)	
Sex		
Male	21	87.5
Female	3	12.5

**Table 2:** Candidate target genes regulated by *miR-143-5p.*

Entrez Gene	Gene Symbol	Gene Name	Total Sites	GSE19188 FC (log_2_)	A549 *miR-143-5p* Transfectant FC (log_2_)	TCGA OncoLnc 5-Year OS *p*-Value
4173	*MCM4*	minichromosome maintenance complex component 4	4	3.1253	−1.0075	0.0025
8091	*HMGA2*	high mobility group AT-hook 2	3	2.8253	−2.5651	0.1389
5888	*RAD51*	RAD51 recombinase	3	2.0850	−1.0812	0.0478
374393	*FAM111B*	family with sequence similarity 111, member B	2	2.0754	−1.8781	0.0003
1047	*CLGN*	calmegin	1	1.8038	−1.1616	0.0362
51155	*HN1*	hematological and neurological expressed 1	1	1.6878	−3.2334	0.4579
222962	*SLC29A4*	solute carrier family 29 (equilibrative nucleoside transporter), member 4	3	1.6166	−1.0253	0.9072
144501	*KRT80*	keratin 80	1	1.4711	−1.4127	0.0093
2817	*GPC1*	glypican 1	1	1.4008	−1.1491	0.0322
9633	*MTL5*	metallothionein-like 5, testis-specific (tesmin)	1	1.4001	−1.0459	0.0001
81831	*NETO2*	neuropilin (NRP) and tolloid (TLL)-like 2	1	1.3829	−2.5034	0.0344
2175	*FANCA*	Fanconi anemia, complementation group A	5	1.3767	−1.1330	0.0218
771	*CA12*	carbonic anhydrase XII	2	1.2698	−1.5449	0.2834
84524	*ZC3H8*	zinc finger CCCH-type containing 8	3	1.2393	−1.1569	0.9583
6929	*TCF3*	transcription factor 3	2	1.2092	−1.3071	0.1287
9650	*MTFR1*	mitochondrial fission regulator 1	1	1.2079	−1.4214	0.0038
29993	*PACSIN1*	protein kinase C and casein kinase substrate in neurons 1	1	1.1587	−2.3003	0.3508
23097	*CDK19*	cyclin-dependent kinase 19	1	1.1498	−1.4269	0.8145
1869	*E2F1*	E2F transcription factor 1	1	1.1114	−1.1265	0.38
10160	*FARP1*	FERM, RhoGEF (ARHGEF) and pleckstrin domain protein 1 (chondrocyte-derived)	1	1.0674	−1.0327	0.0699
23170	*TTLL12*	tubulin tyrosine ligase-like family, member 12	4	1.0180	−1.1575	0.0127
84419	*C15orf48*	chromosome 15 open reading frame 48	2	1.0106	−1.0710	0.134

Lower and upper percentiles of The Cancer Genome Atlas (TCGA) database were both 50. GSE: Gene Expression. Omnibus dataset results; FC: fold change; OS: overall survival.

## Supplementary Materials

Supplementary materials can be found at https://www.mdpi.com/1422-0067/20/18/4482/s1.

## References

[1] Bray F., Ferlay J., Soerjomataram I., Siegel R.L., Torre L.A., Jemal A. (2018). Global cancer statistics 2018: GLOBOCAN estimates of incidence and mortality worldwide for 36 cancers in 185 countries. CA Cancer J. Clin..

[2] Travis W.D. (2011). Pathology of lung cancer. Clin. Chest Med..

[3] Rosell R., Moran T., Queralt C., Porta R., Cardenal F., Camps C., Majem M., Lopez-Vivanco G., Isla D., Provencio M. (2009). Screening for epidermal growth factor receptor mutations in lung cancer. N. Engl. J. Med..

[4] Rosell R., Carcereny E., Gervais R., Vergnenegre A., Massuti B., Felip E., Palmero R., Garcia-Gomez R., Pallares C., Sanchez J.M. (2012). Erlotinib versus standard chemotherapy as first-line treatment for European patients with advanced EGFR mutation-positive non-small-cell lung cancer (EURTAC): A multicentre, open-label, randomised phase 3 trial. Lancet Oncol..

[5] Alkan A., Koksoy E.B., Utkan G. (2015). First-line crizotinib in ALK-positive lung cancer. N. Engl. J. Med..

[6] Joo J.W., Hong M.H., Shim H.S. (2018). Clinical characteristics of T790M-positive lung adenocarcinoma after resistance to epidermal growth factor receptor-tyrosine kinase inhibitors with an emphasis on brain metastasis and survival. Lung Cancer.

[7] Baer C., Claus R., Plass C. (2013). Genome-wide epigenetic regulation of miRNAs in cancer. Cancer Res..

[8] Bartel D.P. (2004). MicroRNAs: Genomics, biogenesis, mechanism, and function. Cell.

[9] Friedman R.C., Farh K.K., Burge C.B., Bartel D.P. (2009). Most mammalian mRNAs are conserved targets of microRNAs. Genome Res..

[10] Nelson K.M., Weiss G.J. (2008). MicroRNAs and cancer: Past, present, and potential future. Mol. Cancer Ther..

[11] Lin S., Gregory R.I. (2015). MicroRNA biogenesis pathways in cancer. Nat. Rev. Cancer.

[12] Goto Y., Kojima S., Kurozumi A., Kato M., Okato A., Matsushita R., Ichikawa T., Seki N. (2016). Regulation of E3 ubiquitin ligase-1 (WWP1) by microRNA-452 inhibits cancer cell migration and invasion in prostate cancer. Br. J. Cancer.

[13] Toda H., Kurozumi S., Kijima Y., Idichi T., Shinden Y., Yamada Y., Arai T., Maemura K., Fujii T., Horiguchi J. (2018). Molecular pathogenesis of triple-negative breast cancer based on microRNA expression signatures: Antitumor miR-204-5p targets AP1S3. J. Hum. Genet..

[14] Fukuhisa H., Seki N., Idichi T., Kurahara H., Yamada Y., Toda H., Kita Y., Kawasaki Y., Tanoue K., Mataki Y. (2019). Gene regulation by antitumor miR-130b-5p in pancreatic ductal adenocarcinoma: The clinical significance of oncogenic EPS8. J. Hum. Genet..

[15] Sugawara S., Yamada Y., Arai T., Okato A., Idichi T., Kato M., Koshizuka K., Ichikawa T., Seki N. (2018). Dual strands of the miR-223 duplex (miR-223-5p and miR-223-3p) inhibit cancer cell aggressiveness: Targeted genes are involved in bladder cancer pathogenesis. J. Hum. Genet..

[16] Gregory R.I., Chendrimada T.P., Cooch N., Shiekhattar R. (2005). Human RISC couples microRNA biogenesis and posttranscriptional gene silencing. Cell.

[17] Mah S.M., Buske C., Humphries R.K., Kuchenbauer F. (2010). miRNA*: A passenger stranded in RNA-induced silencing complex?. Crit. Rev. Eukaryot. Gene Expr..

[18] Uchida A., Seki N., Mizuno K., Misono S., Yamada Y., Kikkawa N., Sanada H., Kumamoto T., Suetsugu T., Inoue H. (2019). Involvement of dual-strand of the miR-144 duplex and their targets in the pathogenesis of lung squamous cell carcinoma. Cancer Sci..

[19] Mataki H., Seki N., Mizuno K., Nohata N., Kamikawaji K., Kumamoto T., Koshizuka K., Goto Y., Inoue H. (2016). Dual-strand tumor-suppressor microRNA-145 (miR-145-5p and miR-145-3p) coordinately targeted MTDH in lung squamous cell carcinoma. Oncotarget.

[20] Misono S., Seki N., Mizuno K., Yamada Y., Uchida A., Arai T., Kumamoto T., Sanada H., Suetsugu T., Inoue H. (2018). Dual strands of the miR-145 duplex (miR-145-5p and miR-145-3p) regulate oncogenes in lung adenocarcinoma pathogenesis. J. Hum. Genet..

[21] Misono S., Seki N., Mizuno K., Yamada Y., Uchida A., Sanada H., Moriya S., Kikkawa N., Kumamoto T., Suetsugu T. (2019). Molecular Pathogenesis of Gene Regulation by the miR-150 Duplex: miR-150-3p Regulates TNS4 in Lung Adenocarcinoma. Cancers (Basel).

[22] Wu X., Piper-Hunter M.G., Crawford M., Nuovo G.J., Marsh C.B., Otterson G.A., Nana-Sinkam S.P. (2009). MicroRNAs in the pathogenesis of Lung Cancer. J. Thorac. Oncol..

[23] Chen X., Xiong D., Yang H., Ye L., Mei S., Wu J., Chen S., Shang X., Wang K., Huang L. (2019). Long noncoding RNA OPA-interacting protein 5 antisense transcript 1 upregulated SMAD3 expression to contribute to metastasis of cervical cancer by sponging *miR-143-3p*. J. Cell. Physiol..

[24] Xia C., Yang Y., Kong F., Kong Q., Shan C. (2018). *MiR-143-3p* inhibits the proliferation, cell migration and invasion of human breast cancer cells by modulating the expression of MAPK7. Biochimie.

[25] Han L., Tang M., Xu X., Jiang B., Wei Y., Qian H., Lu X. (2019). *MiR-143-3p* suppresses cell proliferation, migration, and invasion by targeting Melanoma-Associated Antigen A9 in laryngeal squamous cell carcinoma. J. Cell. Biochem..

[26] Sachdeva M., Zhu S., Wu F., Wu H., Walia V., Kumar S., Elble R., Watabe K., Mo Y.Y. (2009). p53 represses c-Myc through induction of the tumor suppressor miR-145. Proc. Natl. Acad. Sci. USA.

[27] Kojima S., Enokida H., Yoshino H., Itesako T., Chiyomaru T., Kinoshita T., Fuse M., Nishikawa R., Goto Y., Naya Y. (2014). The tumor-suppressive microRNA-143/145 cluster inhibits cell migration and invasion by targeting GOLM1 in prostate cancer. J. Hum. Genet..

[28] Yoshino H., Enokida H., Itesako T., Kojima S., Kinoshita T., Tatarano S., Chiyomaru T., Nakagawa M., Seki N. (2013). Tumor-suppressive microRNA-143/145 cluster targets hexokinase-2 in renal cell carcinoma. Cancer Sci..

[29] Chen J., Chen X. (2018). MYBL2 Is Targeted by *miR-143-3p* and Regulates Breast Cancer Cell Proliferation and Apoptosis. Oncol. Res..

[30] Goto Y., Kurozumi A., Arai T., Nohata N., Kojima S., Okato A., Kato M., Yamazaki K., Ishida Y., Naya Y. (2017). Impact of novel miR-145-3p regulatory networks on survival in patients with castration-resistant prostate cancer. Br. J. Cancer.

[31] Yamada Y., Koshizuka K., Hanazawa T., Kikkawa N., Okato A., Idichi T., Arai T., Sugawara S., Katada K., Okamoto Y. (2018). Passenger strand of miR-145-3p acts as a tumor-suppressor by targeting MYO1B in head and neck squamous cell carcinoma. Int. J. Oncol..

[32] Shimonosono M., Idichi T., Seki N., Yamada Y., Arai T., Arigami T., Sasaki K., Omoto I., Uchikado Y., Kita Y. (2019). Molecular pathogenesis of esophageal squamous cell carcinoma: Identification of the antitumor effects of miR1453p on gene regulation. Int. J. Oncol..

[33] Wu X.L., Cheng B., Li P.Y., Huang H.J., Zhao Q., Dan Z.L., Tian D.A., Zhang P. (2013). MicroRNA-143 suppresses gastric cancer cell growth and induces apoptosis by targeting COX-2. World J. Gastroenterol..

[34] He M., Zhan M., Chen W., Xu S., Long M., Shen H., Shi Y., Liu Q., Mohan M., Wang J. (2017). *MiR-143-5p* Deficiency Triggers EMT and Metastasis by Targeting HIF-1alpha in Gallbladder Cancer. Cell. Physiol. Biochem..

[35] Jin X., Chen X., Hu Y., Ying F., Zou R., Lin F., Shi Z., Zhu X., Yan X., Li S. (2017). LncRNA-TCONS_00026907 is involved in the progression and prognosis of cervical cancer through inhibiting *miR-143-5p*. Cancer Med.

[36] Wu F., Gao H., Liu K., Gao B., Ren H., Li Z., Liu F. (2019). The lncRNA ZEB2-AS1 is upregulated in gastric cancer and affects cell proliferation and invasion via *miR-143-5p*/HIF-1alpha axis. Onco Targets Ther..

[37] Liu C., Wang J.O., Zhou W.Y., Chang X.Y., Zhang M.M., Zhang Y., Yang X.H. (2019). Long non-coding RNA LINC01207 silencing suppresses AGR2 expression to facilitate autophagy and apoptosis of pancreatic cancer cells by sponging *miR-143-5p*. Mol. Cell. Endocrinol..

[38] Grzegrzolka J., Gomulkiewicz A., Olbromski M., Glatzel-Plucinska N., Piotrowska A., Ratajczak-Wielgomas K., Rzechonek A., Podhorska-Okolow M., Krawczuk Z., Dziegiel P. (2019). Expression of tesmin (MTL5) in nonsmall cell lung cancer: A preliminary study. Oncol. Rep..

[39] Sun H., Liu K., Huang J., Sun Q., Shao C., Luo J., Xu L., Shen Y., Ren B. (2019). FAM111B, a direct target of p53, promotes the malignant process of lung adenocarcinoma. Onco Targets Ther..

[40] Santosa V., Martha S., Hirose N., Tanaka K. (2013). The fission yeast minichromosome maintenance (MCM)-binding protein (MCM-BP), Mcb1, regulates MCM function during prereplicative complex formation in DNA replication. J. Biol. Chem..

[41] Liu Y.Z., Wang B.S., Jiang Y.Y., Cao J., Hao J.J., Zhang Y., Xu X., Cai Y., Wang M.R. (2017). MCMs expression in lung cancer: Implication of prognostic significance. J. Cancer.

[42] Wu W., Wang X., Shan C., Li Y., Li F. (2018). Minichromosome maintenance protein 2 correlates with the malignant status and regulates proliferation and cell cycle in lung squamous cell carcinoma. Onco Targets Ther..

[43] Fei L., Ma Y., Zhang M., Liu X., Luo Y., Wang C., Zhang H., Zhang W., Han Y. (2017). RACK1 promotes lung cancer cell growth via an MCM7/RACK1/ Akt signaling complex. Oncotarget.

[44] Jin Y., Xiong A., Zhang Z., Li S., Huang H., Yu T.T., Cao X., Cheng S.Y. (2014). MicroRNA-31 suppresses medulloblastoma cell growth by inhibiting DNA replication through minichromosome maintenance 2. Oncotarget.

[45] Afanasyeva E.A., Mestdagh P., Kumps C., Vandesompele J., Ehemann V., Theissen J., Fischer M., Zapatka M., Brors B., Savelyeva L. (2011). MicroRNA miR-885-5p targets CDK2 and MCM5, activates p53 and inhibits proliferation and survival. Cell Death Differ..

[46] Wang D., Wang H., Li Y., Li Q. (2018). MiR-362-3p functions as a tumor suppressor through targeting MCM5 in cervical adenocarcinoma. Biosci. Rep..

[47] Bai G., Smolka M.B., Schimenti J.C. (2016). Chronic DNA Replication Stress Reduces Replicative Lifespan of Cells by TRP53-Dependent, microRNA-Assisted MCM2-7 Downregulation. PLoS Genet..

[48] Uchida A., Seki N., Mizuno K., Yamada Y., Misono S., Sanada H., Kikkawa N., Kumamoto T., Suetsugu T., Inoue H. (2019). Regulation of KIF2A by Antitumor miR-451a Inhibits Cancer Cell Aggressiveness Features in Lung Squamous Cell Carcinoma. Cancers (Basel).

