# Supplementary material for: Involvement of Dual Strands of miR-143 (miR-143-5p and miR-143-3p) and Their Target Oncogenes in the Molecular Pathogenesis of Lung Adenocarcinoma
Source: Int J Mol Sci. 2019 Sep 11;20(18):4482. doi: 10.3390/ijms20184482 (PMC6770575; doi:10.3390/ijms20184482)
Supplement: Supplementary file 1 [file ijms-20-04482-s001.pdf]

International Journal of *Molecular Sciences*

MDPI

Supplementary Materials: Involvement of Dual Strands of *miR-143* (*miR-143-5p* and *miR-143-3p*) and Their Target Oncogenes in the Molecular Pathogenesis of Lung Adenocarcinoma

Hiroki Sanada, Naohiko Seki, Keiko Mizuno, Shunsuke Misono, Akifumi Uchida, Yasutaka Yamada, Shogo Moriya, Naoko Kikkawa, Kentaro Machida, Tomohiro Kumamoto, Takayuki Suetsugu, and Hiromasa Inoue

Table S1 . Characteristics of patients with LUAD and noncancerous controls.

A. Characteristics of patients with LUAD

Patient no.	Age	Sex	T	N	M	Pathological stage	Pathology
1	75	F	2a	1	0	IIA	LUAD
2	80	F	2	2	0	IIIA	LUAD
3	75	M	2a	1	0	IIA	LUAD
4	59	F	2a	1	0	IIA	LUAD
5	57	M	2	0	0	IIA	LUAD
6	73	F	1b	0	0	IA	LUAD
7	86	F	2a	0	0	IB	LUAD
8	82	M	2a	0	0	IB	LUAD
9	68	M	2a	1	0	IIA	LUAD
10	64	F	2a	2	0	IIIA	LUAD
11	74	M	2a	0	0	IB	LUAD
12	70	M	2b	2	0	IIIA	LUAD
13	72	F	2a	1	0	IIA	LUAD
14	78	M	2a	1	0	IIA	LUAD
15	77	F	2a	1	0	IIA	LUAD
16	74	M	2b	1	0	IIA	LUAD
17	64	M	3	0	0	IIB	LUAD
18	70	F	2a	0	0	IB	LUAD
19	73	M	3	2	0	IIIA	LUAD

B. Characteristics of controls

Patient no.	Age	Sex
1	75	F
2	80	F

3	75	M
4	59	F
20	69	M
21	78	M
22	79	M
23	75	M
24	50	M
25	74	M
26	73	M
27	64	M
28	88	M
29	68	M
30	72	M
31	76	M
32	73	M
33	76	M
34	65	M
35	69	M
36	50	M
37	65	M
38	69	M
39	65	M

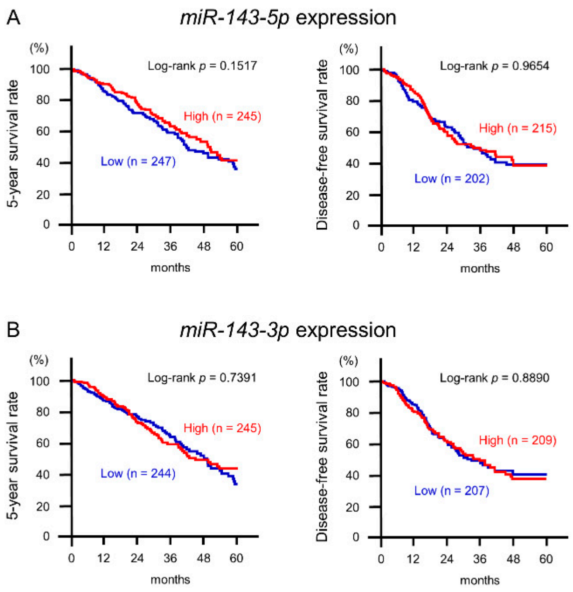

Figure S1. Analyses of the expression levels of *miR-143-5p* and *miR-143-3p* and the clinical features of patients with LUAD using data obtained from TCGA database. Clinical significance was not detected in Kaplan–Meier curves of 5-year overall survival and 5-year disease-free survival.

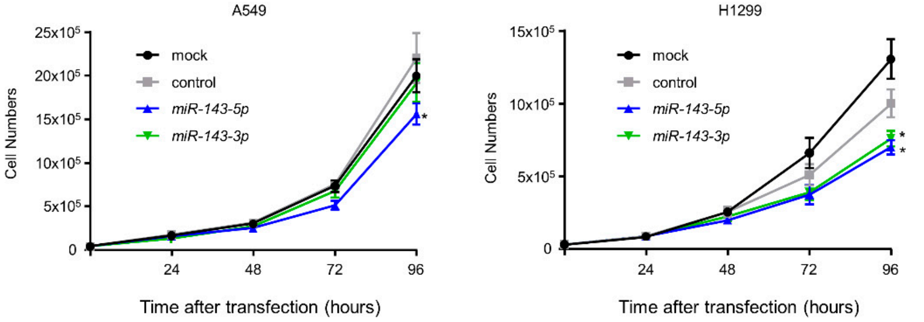

Figure S2. Cell proliferation assay by using cell counting LUAD cells with ectopic expression of *miR-143-5p* and *miR-143-3p*. The number of cells was counted at 24, 48, 72, 96 h after transfection and the proliferation abilities were evaluated. In H1299 cells, cell growth decreased at 96 h after transfection compared to controls. In A549 cells, cell growth was decreased only in *miR-143-5p* transfected cells (**p* < 0.001).

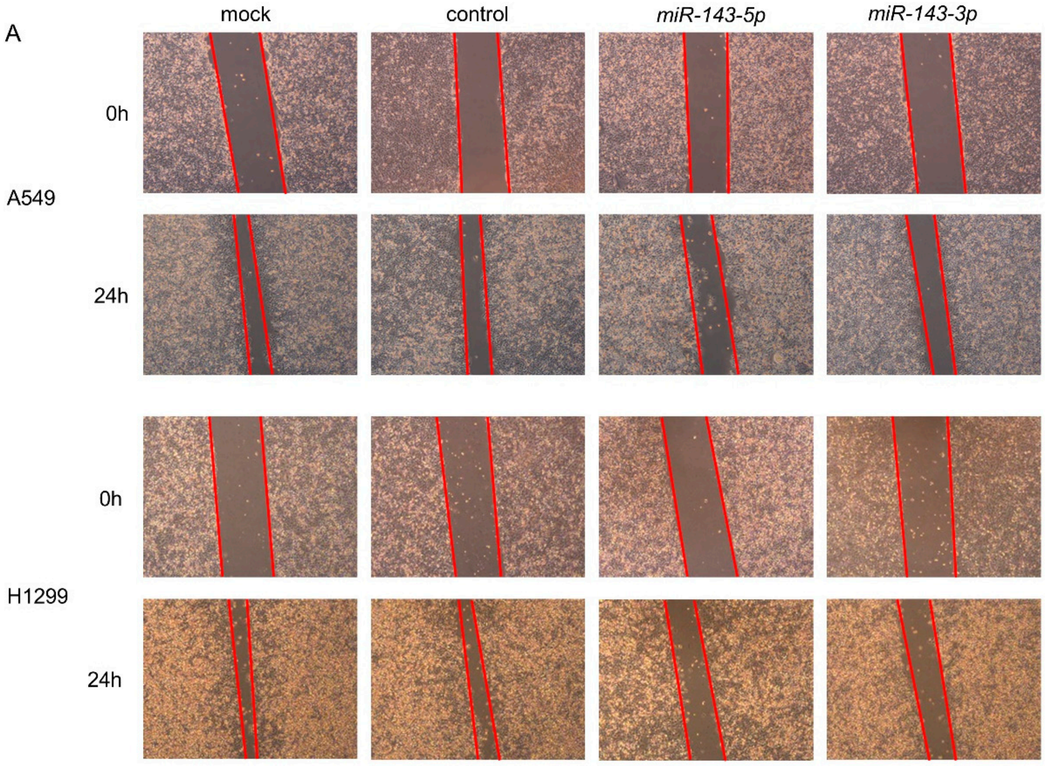

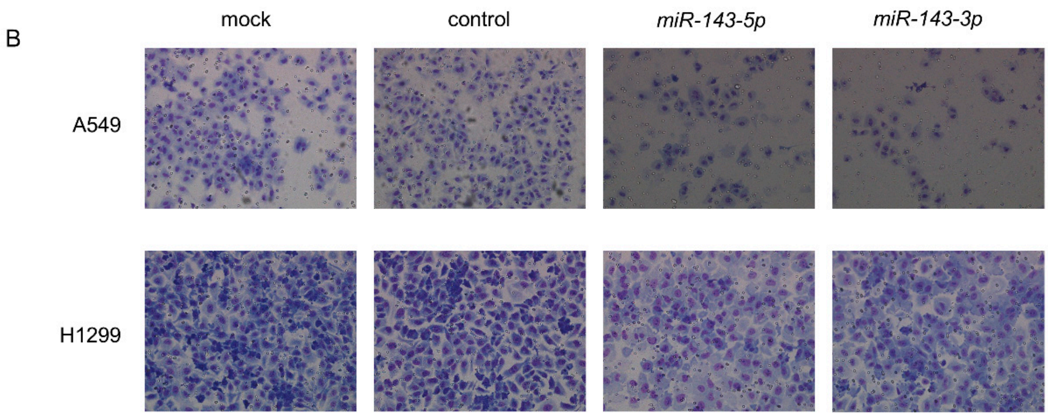

Figure S3. Cell migration and invasion assays in LUAD cells with ectopic expression of *miR-143-5p* and *miR-143-3p*. (**A**) Phase-contrast micrographs of LUAD cells with *miR-143-5p* and *miR-143-3p* transfected in migration assay are shown. (**B**) Micrographs of LUAD cells with *miR-143-5p* and *miR-143-3p* transfected in Matrigel invasion assays are shown.

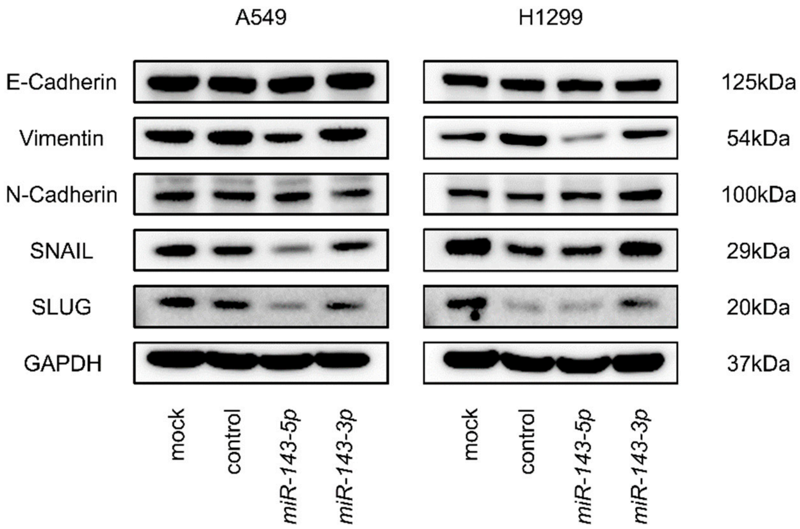

Figure S4. Expression of EMT-related proteins regulated by *miR-143-5p* and *miR-143-3p* in LUAD cells. After *miR-143-5p* and *miR-143-3p* was transfected into A549, the expression of EMT-related proteins was confirmed by Western blotting. The mesenchymal markers Vimentin, SNAIL, and SLUG was suppressed with *miR-143-5p*.

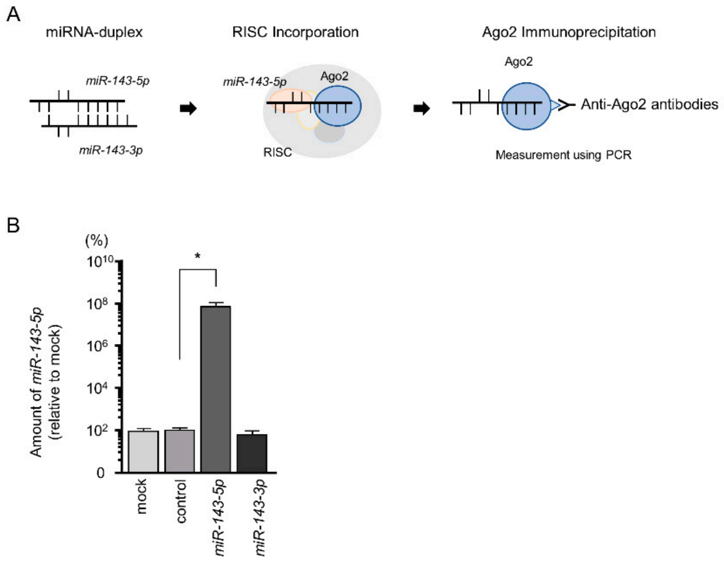

Figure S5. Incorporation of *miR-143-5p* into the RISC in LUAD cells. To investigate the incorporation of the passenger strand of miRNA into the RISC, immunoprecipitation with anti-Ago2 antibodies was performed after *miR-143-5p* transfection into A549 cells. (**A**) A schematic diagram of the isolation of RISC-incorporated *miR-143-5p* by Ago2 immunoprecipitation is shown. (**B**) The amount of *miR-143-5p* was markedly elevated by *miR-143-5p* transfection into LUAD cells (*$p < 0.001$).

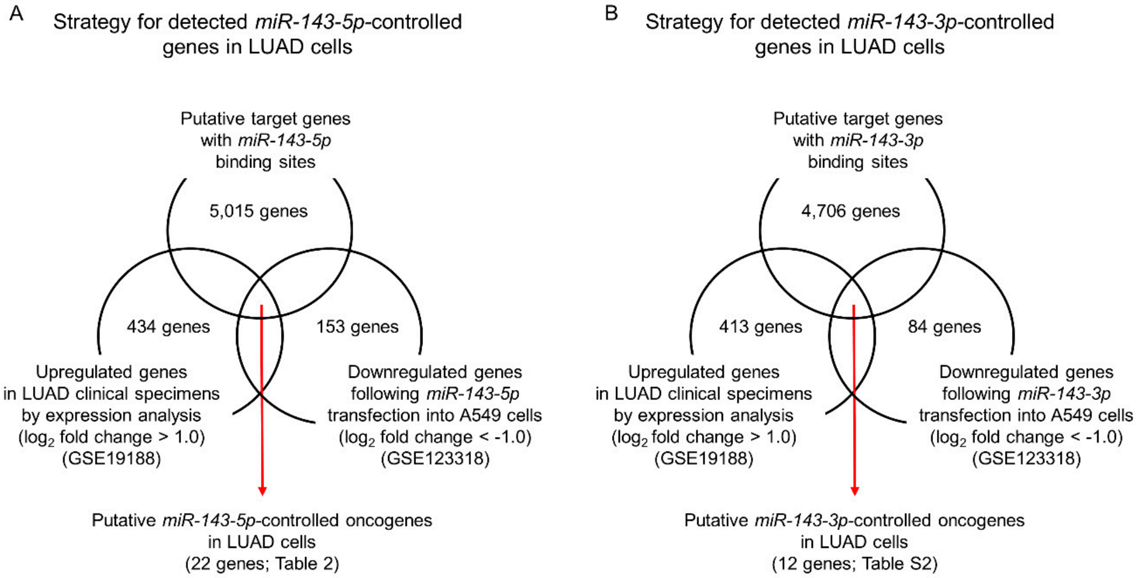

Figure S6. Strategy for identification of (**A**) *miR-143-5p* and (**B**) *miR-143-3p* target oncogenes in LUAD cells.

Table S2. Candidate target genes regulated by *miR-143-3p*.

Entrez Gene	Gene Symbol	Gene Name	Total Sites	GSE19188 FC (log_2)	A549 *miR-143-3p* Transfectant FC (log_2)	TCGA OncoLnc 5-Year OS *p*- value
1058	*CENPA*	centromere protein A	1	3.488	-1.056	0.0365
4173	*MCM4*	minichromosome maintenance complex component 4	2	3.125	-1.096	0.0025
124540	*MSI2*	musashi RNA-binding protein 2	3	2.120	-1.249	0.9231
92312	*MEX3A*	mex-3 RNA binding family member A	2	1.986	-1.250	0.1271
221833	*SP8*	Sp8 transcription factor	1	1.731	-1.002	no date
55055	*ZWILCH*	zwilch kinetochore protein	1	1.534	-1.684	0.0148
65263	*PYCRL*	pyrroline-5-carboxylate reductase-like	1	1.491	-1.198	0.9189
144501	*KRT80*	keratin 80	1	1.471	-1.393	0.0093
6624	*FSCN1*	fascin homolog 1, actin-bundling protein (Strongylocentrotus purpuratus)	2	1.443	-1.108	0.0017
143503	*OR51E1*	olfactory receptor, family 51, subfamily E, member 1	1	1.335	-1.417	0.0326
165545	*DQX1*	DEAQ box RNA-dependent ATPase 1	1	1.316	-1.190	0.2973
655	*BMP7*	bone morphogenetic protein 7	1	1.050	-1.051	0.6734

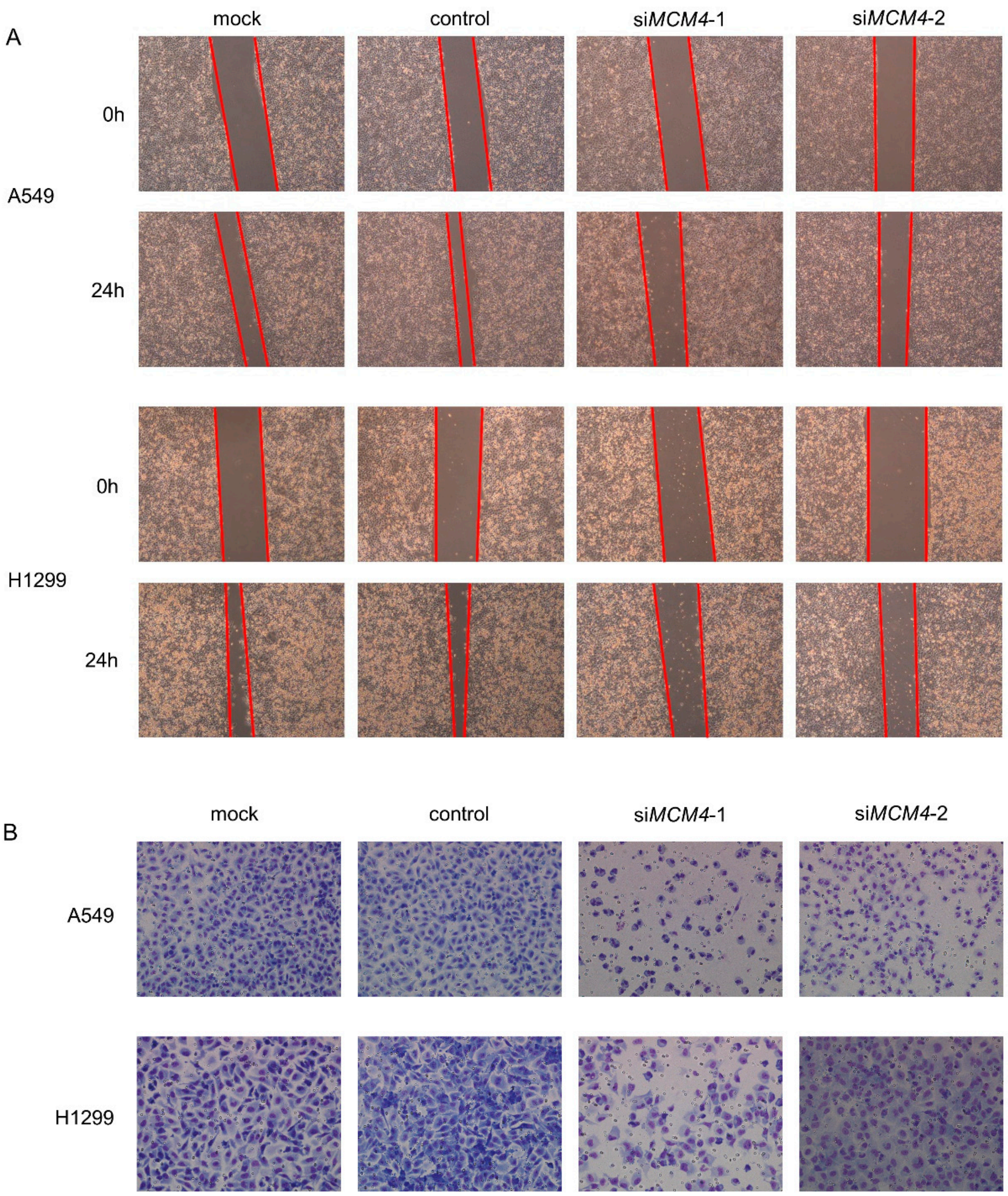

Figure S7. Cell migration and invasion assays in LUAD cells with ectopic expression of si*MCM4*. (**A**) Phase-contrast micrographs of LUAD cells with si*MCM4* transfected in migration assay are shown. (**B**) Micrographs of LUAD cells with si*MCM4* transfected in Matrigel invasion assays are shown.

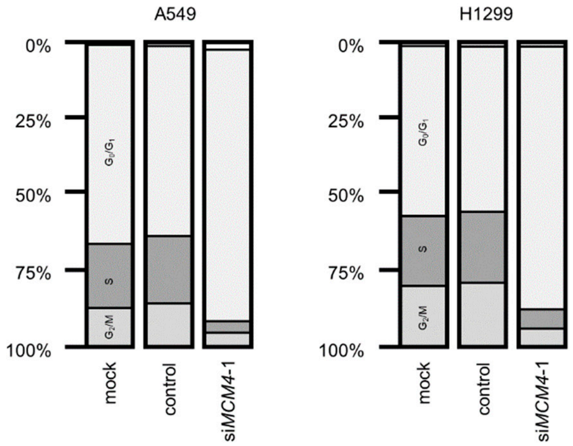

Figure S8. Effects of *MCM4* silencing in cell cycle assay in LUAD cells. G_0/G_1 phase arrest was observed after si*MCM4*-1 transfection into LUAD cells.

Table S3. Immunohistochemical status and characteristics of the LUAD and noncancerous cases.

A. Immunohistochemical status and characteristics of LUAD cases

Patient no.	T	N	M	Pathological stage	Immunohistochemical intensity	Immunohistochemical extensity
1	1	0	0	IA	2	1
2	2	1	0	IIA	1	1
3	1	0	0	I	2	1
4	2	2	0	IIIA	0	0
5	3	1	0	IIIA	0	0
6	2	0	0	IB	0	0
7	3	2	0	IIIA	1	1
8	1	0	0	IA	3	3
9	3	1	0	IIIA	3	1
10	4	0	0	IIIA	3	3
11	2	0	0	IIA	0	0
12	3	1	0	IIIA	0	0
13	2	1	0	IIB	0	0
14	3	0	0	IIB	0	0
15	3	1	0	IIIA	1	1
16	2	1	0	IIB	3	3
17	2	0	0	IB	1	1
18	2	0	0	IB	1	2
19	2	0	0	IB	3	3
20	2	1	0	IIB	2	2

B. Immunohistochemical status of noncancerous cases

Patient no.	Immunohistochemical intensity	Immunohistochemical extensity
21	0	0
22	0	0
23	1	1
24	0	0
25	1	1
26	0	1
27	0	0
28	1	1
29	1	1
30	0	0
31	1	1
32	1	2
33	0	0
34	0	0

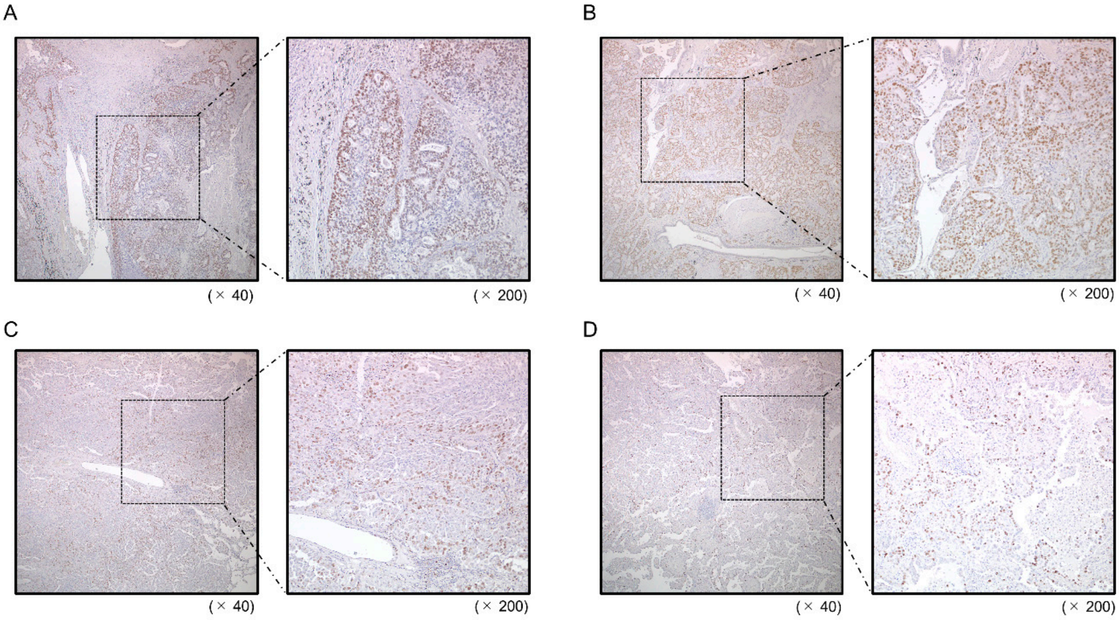

Figure S9. Immunohistochemical staining of MCM4 protein in LUAD. Patient characteristics showed in Table S1. (**A**) patient no. 19. (**B**) patient no. 9. (**C**) patient no.2. (**D**) patient no. 3.

Table S4. Reagents used in this study.

Antibody	Dilution	Catalog number	Company
Anti-MCM4	IHC 1:2000<br>WB 1:5000	ab4459	Abcam, Cambridge, UK
Anti-GAPDH	WB 1:20000	MAB374	EMD Millipore, Billerica, MA, USA
Anti-E Cadherin	WB 1:500	ab15148	Abcam, Cambridge, UK
Anti-N Cadherin	WB 1:1000	ab18203	Abcam, Cambridge, UK
Anti-Vimentin	WB 1:1000	ab8978	Abcam, Cambridge, UK
Anti-SNAIL	WB 1:500	ab53519	Abcam, Cambridge, UK
Anti-SLUG	WB 1:500	ab27568	Abcam, Cambridge, UK
**miRNA species**	**Concentration**	**Assay ID**	**Company**
miR-143-5p	10 nM	PM 12540	Applied Biosystems, Foster City, CA, USA
miR-143-3p	10 nM	PM 10883	Applied Biosystems, Foster City, CA, USA
anti-miR Negative Control #1	10 nM	AM 17010	Applied Biosystems, Foster City, CA, USA
**siRNA species**	**Concentration**	**Catalog number**	**Company**
si*MCM4*-1	10 nM	HSS106396	Invitrogen, Carlsbad, CA, USA
si*MCM4*-2	10 nM	HSS106397	Invitrogen, Carlsbad, CA, USA
**Primer and probe**		**Assay ID**	**Company**
miR-26a		000405	Applied Biosystems, Foster City, CA, USA
miR-143-5p		002146	Applied Biosystems, Foster City, CA, USA
miR-143-3p		002249	Applied Biosystems, Foster City, CA, USA
RNU48		001006	Applied Biosystems, Foster City, CA, USA
MCM4		Hs00907398_m1	Applied Biosystems, Foster City, CA, USA
GAPDH		Hs99999905_m1	Applied Biosystems, Foster City, CA, USA
RAD51		Hs00947967_m1	Applied Biosystems, Foster City, CA, USA
FAM111B		Hs01941351_s1	Applied Biosystems, Foster City, CA, USA
CLGN		Hs01567715_m1	Applied Biosystems, Foster City, CA, USA
KRT80		Hs01372365_m1	Applied Biosystems, Foster City, CA, USA
GPC1		Hs00892476_m1	Applied Biosystems, Foster City, CA, USA
MTL5		Hs01127478_m1	Applied Biosystems, Foster City, CA, USA
NETO2		Hs00983147_g1	Applied Biosystems, Foster City, CA, USA
FANCA		Hs01116668_m1	Applied Biosystems, Foster City, CA, USA
MTFR1		Hs00206110_m1	Applied Biosystems, Foster City, CA, USA
TTLL12		Hs00209450_m1	Applied Biosystems, Foster City, CA, USA

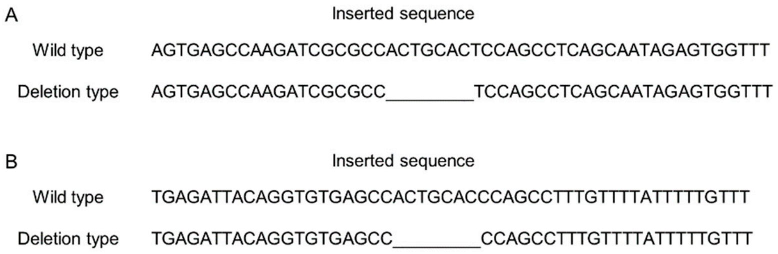

Figure S10. Sequences inserted into the vector for dual-luciferase reporter assays. These sequences were cloned into the psiCHECK-2 vector: the wild-type sequence of the 3' untranslated regions (UTRs) of *MCM4* or the deletion type, which lacked the *miR-143-5p* target sites from 3′-UTRs of *MCM4*. (**A**) Position 448–454. (**B**) Position 968–974.

© 2019 by the authors. Submitted for possible open access publication under the terms and conditions of the Creative Commons Attribution (CC BY) license (http://creativecommons.org/licenses/by/4.0/).
